# Development of Shunt-Type Three-Phase Active Power Filter with Novel Adaptive Control for Wind Generators

**DOI:** 10.1155/2015/963456

**Published:** 2015-09-15

**Authors:** Ming-Hung Chen

**Affiliations:** Department of Electrical Engineering, Ming Chi University of Technology, 84 Gungjuan Road, Taishan District, New Taipei City 24301, Taiwan

## Abstract

This paper proposes a new adaptive filter for wind generators that combines instantaneous reactive power compensation technology and current prediction controller, and therefore this system is characterized by low harmonic distortion, high power factor, and small DC-link voltage variations during load disturbances. The performance of the system was first simulated using MATLAB/Simulink, and the possibility of an adaptive digital low-pass filter eliminating current harmonics was confirmed in steady and transient states. Subsequently, a digital signal processor was used to implement an active power filter. The experimental results indicate, that for the rated operation of 2 kVA, the system has a total harmonic distortion of current less than 5.0% and a power factor of 1.0 on the utility side. Thus, the transient performance of the adaptive filter is superior to the traditional digital low-pass filter and is more economical because of its short computation time compared with other types of adaptive filters.

## 1. Introduction

Generally, wind generators are connected with grid for providing electrical power from wind energy. It is known that power electronic equipment is widely used in industrial processes and automatic machines for promotion of motor performance. Consequently, the traditional rectifiers, diode-bridge rectifiers, or phase-controlled rectifiers which are largely used to provide dc source for the inverters cause the serious harmonic problem of power quality. These current harmonics result in problems such as a low power factor, low efficiency, power system voltage fluctuations, and communication interference [1]. Using an appropriate power filter for improving power quality is the solution to these problems. There are two types of power filters: passive [2] and active [3–5]. A passive power filter (PPF) consists of inductors and capacitors. The circuit design of a PPF is simple, but it has the following disadvantages: (1) Each PPF is suitable only for a specific harmonic, unless the harmonics are sufficiently high or low to be eliminated; designing a group of passive filters for various harmonics is uneconomical. (2) Using a PPF for improving the power factor is less effective, and the original design is not applicable if the system architecture is changed. (3) A PPF may produce series or parallel resonance with the circuit impedance, leading to equipment damage. (4) The source impedance substantially affects the filtering properties of the filter. (5) A low-resistance circuit may generate additional current harmonics, rendering a passive filter ineffective. Finally, (6) a PPF occupies a larger space. Thus, using a PPF to eliminate harmonics is not an effective solution.

An active power filter (APF) is implemented using power switches, inductors, capacitors, and a control circuit, which calculates the compensation current required to eliminate current harmonics for preventing resonance. The disadvantage of the APF is that its switching loss is greater than that of the passive filter. Moreover, the controller of the APF is complicated, resulting in low reliability. However, because of the current level of technological advancement, it is easy to develop microprocessors or power electronics components that can be used in APF implementation for overcoming these disadvantages. Using an APF to eliminate current harmonics will become the development trend of the future. The most critical factor influencing the effectiveness of an APF involves determining the current harmonic, which can be obtained from the frequency or time domain. Frequency domain calculation is based on Fourier analysis, and most time domain approaches entail using instantaneous reactive power theory. The adaptive finite impulse response digital filter [6] was used in this study for reducing the time delay and eliminating the steady state error.

## 2. System Configuration

**Figure 1:**
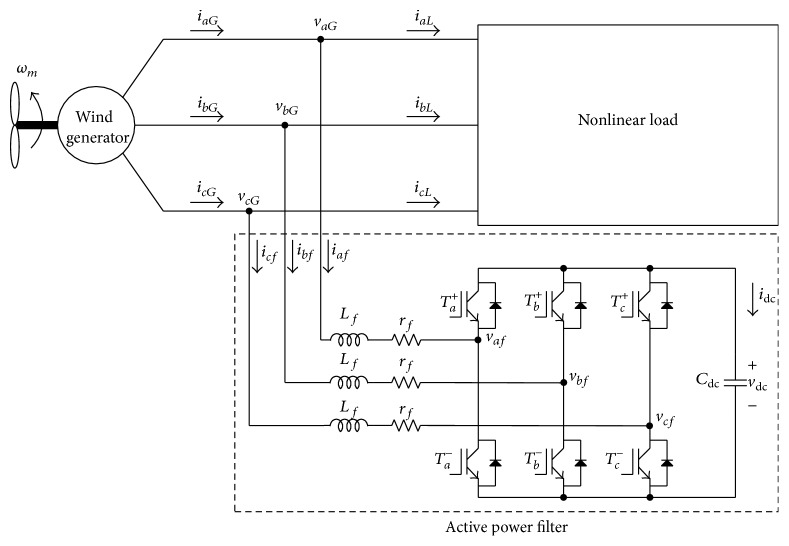
Block diagram of the proposed system.

The proposed shunt-type three-phase APF for wind generators is shown in Figure 1. It consists of boost inductors, a three-phase full-bridge power converter, and a DC-link capacitor; *i*_*af*_, *i*_*bf*_, *i*_*cf*_, *i*_*aL*_, *i*_*bL*_, and *i*_*cL*_ represent the currents provided to the power converter and nonlinear load. The three line currents of wind generator are calculated by the summation of the three line currents to nonlinear load and active power filter. To eliminate current harmonic components generated by the nonlinear load, the APF provides equal but opposite harmonic currents to the point of connection with the nonlinear load. This reduces the original distortion and corrects the power factor. For the sake of simplicity, the reference frame transformation method was used.

## 3. Operation Principles

A simplified analytical model of the APF was derived to analyse the DC-link voltage response and current tracking capability. The application of the proposed control strategy leads to the current harmonics being compensated quickly and the fluctuations in the DC-link voltage during transient and steady states being effectively suppressed.

### 3.1. Mathematical Model of the Active Power Filter

**Figure 2:**
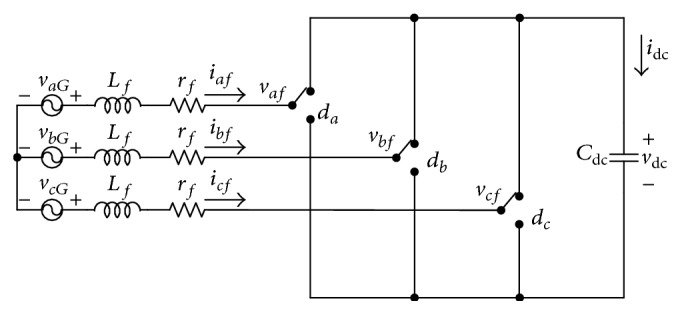
Equivalent circuit of active power filter.

Consider Figure 1, where the dashed box represents the circuit diagram of the APF. If the reactors of the wind generator are neglected, the equivalent circuit of the APF can be obtained as shown in Figure 2. The switch status *d*_*x*_ can be written as

(1) $$\begin{array}{c} d_{x}=\left\{\begin{array}{cc} 1 & \text{if }T_{x}^{+}\text{: on},\;T_{x}^{-}\text{: off}\\ 0 & \text{if }T_{x}^{+}\text{: off},\;T_{x}^{-}\text{: on}, \end{array}\right. \end{array}$$

where *T*_*x*_^+^ and *T*_*x*_^−^ represent power transistors and *x* denotes *a*, *b*, or *c*. The switch voltages *v*_*af*_, *v*_*bf*_, and *v*_*cf*_ and the DC-link voltage *v*_dc_ can be expressed as [7]

(2) $$\begin{array}{c} \left[\begin{array}{c} v_{af}\\ v_{bf}\\ v_{cf} \end{array}\right]=\frac{v_{\text{dc}}}{3}\left[\begin{array}{ccc} 2 & -1 & -1\\ -1 & 2 & -1\\ -1 & -1 & 2 \end{array}\right]\left[\begin{array}{c} d_{a}\\ d_{b}\\ d_{c} \end{array}\right]. \end{array}$$

The differential equations for Figure 2 are as follows:

(3) Lfddtiaf=vaG−rfiaf−vaf,Lfddtibf=vbG−rfibf−vbf,Lfddticf=vcG−rficf−vcf,Cdcddtvdc=daiaf+dbibf+dcicf,

where *v*_*aG*_, *v*_*bG*_, and *v*_*cG*_ represent the phase voltages of the wind generator and *v*_*af*_, *v*_*bf*_, and *v*_*cf*_ represent the input voltages of the three-phase full-bridge power converter; *r*_*f*_ and *L*_*f*_ are the resistance and inductance of the APF line reactors, respectively, and *C*_dc_ is the capacitance of the DC-link capacitor.

For model analysis and controller design, the three-phase voltages, currents, and switching functions can be transformed to* d-q-0* rotating frame, thus yielding

(4) fdfqf0=23sin⁡θesin⁡θe−2π3sin⁡θe+2π3cos⁡θecos⁡θe−2π3cos⁡θe+2π3121212fafbfc,

where *θ*_*e*_ is the transformation angle of the rotating frame and *f* denotes voltages, currents, or switching functions.

Based on (3)-(4), the state model in the rotating frame can be written as

(5) $$\begin{array}{c} \left[\begin{array}{c} v_{dG}\\ v_{qG} \end{array}\right]=\left[\begin{array}{cc} L_{f}p+r_{f} & -\omega_{e}L_{f}\\ \omega_{e}L_{f} & L_{f}+r_{f} \end{array}\right]\left[\begin{array}{c} i_{df}\\ i_{qf} \end{array}\right]+\left[\begin{array}{c} v_{df}\\ v_{qf} \end{array}\right], \end{array}$$

where *p* is the operator *d*/*dt*; *ω*_*e*_ is the frequency of the wind generator, and the subscripts “*d*” and “*q*” are used to denote the components of* d*- and* q*-axis in the rotating frame, respectively. Equation (5) was used to derive the block diagram of the APF and calculate the input voltage commands of the power converter.

### 3.2. Design of Passive Components

Passive components of the APF mainly refer to the boost inductors and DC-link capacitor. From the standpoint of manufacturing capacity, the production cost can be reduced by using passive components with a smaller size and lower weight. From the viewpoint of control, using small-sized boost inductors results in a high rate of change of input currents, which in turn increases the total harmonic distortion of current (THD_*i*_). The main considerations for the DC-link capacitor are the system stability and the DC-link voltage ripple. A lower capacitance of the DC-link capacitor affects the system stability and increases the voltage ripple. Therefore, the design of passive components is necessary for achieving a tradeoff between cost and performance, and it is discussed as follows.

Assume that the system is balanced for the maximum input power under the steady state condition and that the switching frequency of the APF is sufficiently high to render the current harmonic in the system negligible. Under such circumstances, the following inequality for the proper operation of the converter can be derived [8]:

(6) $$\begin{array}{c} v_{\text{dc}}\geq 2v_{m}^{\prime }, \end{array}$$

where $V_{m}^{\prime }=\sqrt{{\left(V_{m}-I_{m}r_{f}\right)}^{2}+{\left(\omega_{e}L_{f}I_{m}\right)}^{2}}$; *V*_*m*_ and *I*_*m*_ are the peak values of the voltage and current from the wind generator, respectively. When the command of the DC-link voltage *V*_dc_^*∗*^ is fixed, the maximum inductance of the boost inductors determined from (6) can be written as

(7) $$\begin{array}{c} L_{f,\max}=\frac{3V_{m}}{2\omega_{e}P_{i}}\sqrt{\frac{{V_{\text{dc}}^{*}}^{2}}{4}-{\left(V_{m}-\frac{2P_{i}r_{f}}{3V_{m}}\right)}^{2}}, \end{array}$$

where *P*_*i*_ is the rated input power of the APF. A lower inductance generates higher current harmonics, and therefore the minimum inductance of the boost inductors is limited by THD_*i*_ of the input currents. The voltage harmonics with sinusoidal pulse-width modulation are obtained as follows [9]:

(8) $$\begin{array}{c} \hat{V_{n}}=h_{n}\frac{V_{\text{dc}}}{2}, \end{array}$$

where *h*_*n*_ denotes the ratio of *n*th-order voltage harmonics $\hat{V_{n}}$ to the average voltage *V*_dc_ of the DC-link. The parameter $\hat{V_{n}}$ decreases when the switching frequency increases. *n*th-order current harmonics $\hat{I_{n}}$ can be derived as follows:

(9) $$\begin{array}{c} \hat{I_{n}}=\frac{\hat{V_{n}}}{n\omega_{e}L_{f}}=\frac{h_{n}V_{\text{dc}}}{2n\omega_{e}L_{f}}. \end{array}$$

According to IEEE 519 and IEC 1000-3, THD_*i*_ should be below 5%. Therefore, the minimum inductance of the boost inductors determined from (9) can be written as

(10) $$\begin{array}{c} L_{f,\min}=\frac{15V_{\text{dc}}^{*}V_{m}}{\omega_{e}P_{i}}\sqrt{\underset{n\neq 1}{\sum }{\left(\frac{h_{n}}{n}\right)}^{2}}. \end{array}$$

The capacitance of the DC-link capacitor affects the DC voltage fluctuations in the transient state and the DC voltage ripple in the steady state. Generally, the DC voltage has a safe operating range. If the voltage is too high, it causes high current harmonics to damage the power transistors of the converter; however, if the voltage is too low, freewheel diodes cannot be reverse-biased to facilitate the current control of the APF. The power control strategy directly affects the DC voltage ripple, and ideal power balance control results in zero average current to the DC-link capacitor, even if there are no DC voltage fluctuations in the transient state. However, reducing the average current to the DC-link capacitor to zero in the transient state is difficult. The DC voltage fluctuation of the DC-link capacitor is inversely proportional to the capacitance but is directly proportional to the current integral of the DC-link capacitor. Therefore, the minimum capacitance of the DC-link capacitor can be determined according to the maximum DC voltage fluctuation:

(11) $$\begin{array}{c} C_{\text{dc,min}}=\frac{t_{1}\int_{0}^{t_{1}}i_{\text{dc}}dt}{\Delta v_{\text{dc,max}}}, \end{array}$$

where *i*_dc_ is the current to the DC-link capacitor; the period from 0 to *t*_1_ and Δ*v*_dc,max_ denote the transient response period and maximum DC voltage fluctuation, respectively. Assume that the input currents of the APF are balanced. The ripple factor *k*_*v*_ of the DC voltage can be expressed as

(12) $$\begin{array}{c} k_{v}=\frac{\sqrt{\sum_{n=0}^{\infty }V_{cn}^{2}}}{V_{\text{dc}}}, \end{array}$$

where *V*_*cn*_ = (*X*_*c*_/*n*)*I*_*cn*_; *V*_*cn*_ and *I*_*cn*_ are the effective values of *n*th-order voltage and the current harmonics of the DC-link capacitor, respectively, and *X*_*c*_ is the capacitance of the DC-link capacitor at 60 Hz. According to (12), the minimum capacitance of the DC-link capacitor can be obtained as follows:

(13) $$\begin{array}{c} C_{\text{dc,min}}=\frac{\sqrt{\sum_{n\neq 1}{\left(I_{cn}/n\right)}^{2}}}{\omega_{e}V_{\text{dc}}^{*}k_{v,\max}}, \end{array}$$

where *k*_*v*,max_ is the maximum allowed ripple factor of the DC-link voltage and is generally below 5% [10]. Equations (7), (10), and (13) were used in the current study to design the boost inductors and DC-link capacitor.

### 3.3. Design of Controllers

**Figure 3:**
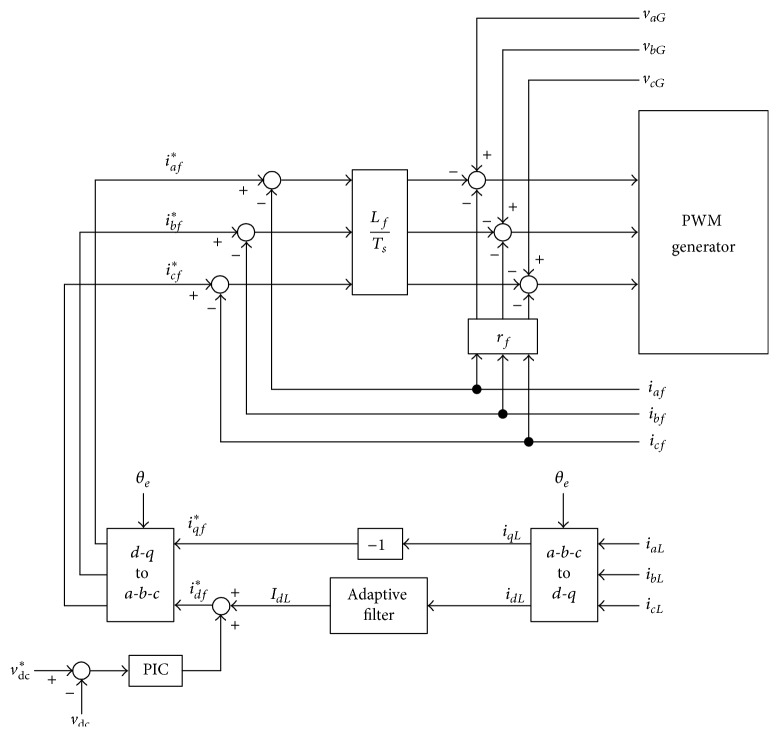
Control block diagram of *d*-*q* current controllers of the active power filter.

The basic principle of the three-phase APF involves generating compensation currents by using a power converter. The compensation currents are injected into the power line to suppress current harmonics and to provide the required power to loads. In the literature, the analytical model of an APF is established under a rotating frame to reduce complexity [11]. Equation (5) shows that the cross-coupling terms *ω*_*e*_ *L*_*f*_ *i*_*df*_ and *ω*_*e*_ *L*_*f*_ *i*_*df*_ exist in *d*-*q* current control loops. To decouple *d*-*q* current loops and simplify the control scheme, voltage decouplers can be implemented using proportional-integral controllers (PIC). The block diagram of *d*-*q* control loops can be derived from (5), as shown in Figure 3. Adaptive control is used in Figure 3 to reduce the time delay of the reference current as well as the DC-link voltage feedback.

**Figure 4:**
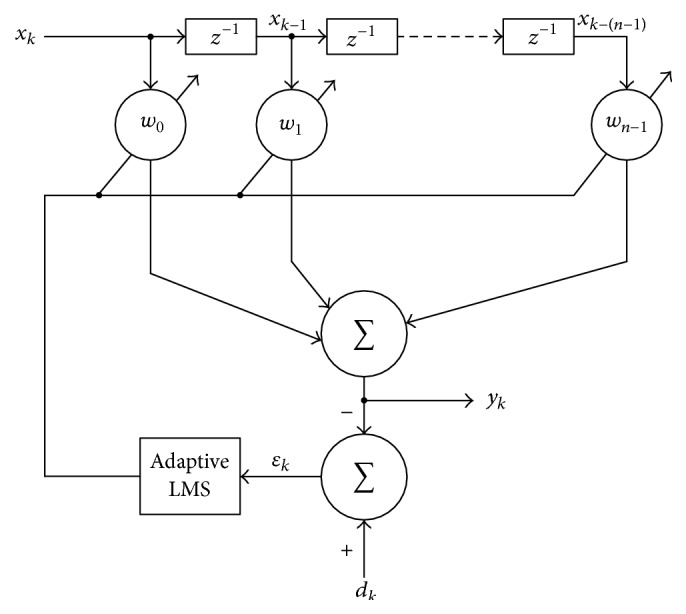
Block diagram of finite impulse response filter.

An adaptive filter is a programmable filter that uses a least mean square (LMS) to calculate errors by subtracting output signals of the filter from expected signals; weighting is used to suppress noise on the input side of the filter [12]. Generally, the adaptive filter uses the finite impulse response technique to stabilize the system response with a single zero of the transfer function shown in Figure 4, but increasingly more time delay elements should be used to achieve higher performance and increase the number of weighting of the filter. The core part of the adaptive filter uses a linear combiner with *n* time delays to generate filter output *y*_*k*_. The adaptive filter with finite impulse response has weighting *w*_0_ to weighting *w*_*n*−1_ to minimize errors from LMS. The calculation of LMS can be expressed as [13]

(14) $$\begin{array}{c} w_{k-1}=w_{k}+2\beta \varepsilon_{k}x_{k}, \end{array}$$

where *w*_*k*_ and *x*_*k*_ denote the weighting and *k*th input, respectively; *ε*_*k*_ is the error obtained by subtracting the output signal of the filter from the expected signal, and *β* is the convergence rate of LMS. According to Figure 4, the expression of the output *y*_*k*_ according to the input *x*_*k*_ is

(15) $$\begin{array}{c} y_{k}=x_{k}^{T}w_{k}=w_{k}^{T}x_{k}, \end{array}$$

where

(16) wk=w0⋯wn−1T,xk=xk⋯xk−n−1T.

*k*th error *ε*_*k*_ can be derived as

(17) $$\begin{array}{c} \varepsilon_{k}=d_{k}-y_{k}=d_{k}-x_{k}^{T}w_{k}, \end{array}$$

where *d*_*k*_ is the expectation response. Based on (17), the following expression can be obtained:

(18) $$\begin{array}{c} \varepsilon_{k}^{2}=d_{k}^{2}-2d_{k}x_{k}^{T}w_{k}+{\left(\;w_{k}^{T}x_{k}\;\right)}^{2}. \end{array}$$

The mean square error (MSE) derived from (18) can be expressed as

(19) $$\begin{array}{c} J≜E\left[\;\varepsilon_{k}^{2}\;\right]=\left[\;d_{k}^{2}\;\right]-2{\overset{-}{P_{x}}}^{T}w_{k}+w_{k}^{T}\overset{-}{R_{x}}w_{k}, \end{array}$$

where $\overset{-}{P_{x}}≜E\left[d_{k}x_{k}\right]$ and $\overset{-}{R_{x}}≜E\left[x_{k}x_{k}^{T}\right]$ denote the cross-correlation matrix and input correlation matrix, respectively. As expressed in (19), *J* is a second-order function weighting. To obtain the minimum MSE, set ∇*J* = 0; that is,

(20) ∇J∂Eεk2∂w0⋯∂Eεk2∂wn−1T=−2Px−+2Rx−wk=0.

Assume that $\overset{-}{R_{x}}$ is a nonsingular matrix. From (20), the Wiener weighting vector *w*_op_^*∗*^, which is an optimized value, can be expressed as

(21) $$\begin{array}{c} w_{\text{op}}^{*}={\overset{-}{R_{x}}}^{-1}\overset{-}{P_{x}}. \end{array}$$

The minimum MSE *J*_min_ can be written as follows by substituting (21) into (19):

(22) $$\begin{array}{c} J_{\min}=E\left[\;d_{k}^{2}\;\right]-2{\overset{-}{P_{x}}}^{T}{\overset{-}{R_{x}}}^{-1}\overset{-}{P_{x}}+{\left[\;{\overset{-}{R_{x}}}^{-1}\overset{-}{P_{x}}\;\right]}^{T}\overset{-}{R_{x}}{\overset{-}{R_{x}}}^{-1}\overset{-}{P_{x}}. \end{array}$$

**Figure 5:**
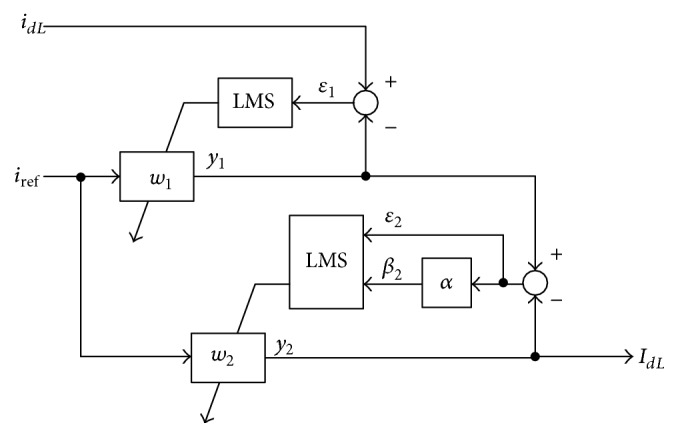
Block diagram of novel adaptive filter.

To accelerate the convergence of the adaptive filter, step size *β* is applied to adjust the weighting [14]. When *β* is large, the time taken for convergence of *w*_*k*_ to *w*_op_^*∗*^ is short. However, it is easy to generate substantial oscillations, and therefore there is a large steady state error. When a smaller *β* is applied, the convergence is slow initially, but the oscillations near *w*_op_^*∗*^ are low. Therefore, the steady state error is smaller. To obtain appropriate *β*, *β* is adjusted using the error as follows:

(23) $$\begin{array}{c} \beta_{k}=\alpha \left|\;\varepsilon_{k}\;\right|, \end{array}$$

where 0 < *α* < 1. Based on (23), the adjusting range is large when the error is large, and *β* decreases when *w*_*k*_ approaches *w*_op_^*∗*^. In this paper, the variable step-size scheme is implemented in series with the conventional adaptive filter. The fixed-step-size MSE is first used to accelerate the transient response, and the variable-step-size MSE is then used to improve the steady state response. The novel adaptive filter shown in Figure 5 can effectively reduce the convergence time compared with the conventional filter. It consists of a calculation circuit for the reference current and two LMS for the relation between reference and real currents of the adaptive filter. Only two coefficients are needed to be adapted in the LMS with two inputs. When any significant changes happen in the load current, the load current is detected and the step size is immediately increased and then decreased slowly until it reaches the initial value. This procedure guarantees a fast transient response to adaptive filter. The adaption procedure is the same as used in the general structure of adaptive filters, and the output signal *I*_*dL*_ tracks the variation of the fundamental signal of the load current. Figure 5 shows that

(24) y1=w1iref,y2=w2iref,

where *i*_ref_ denotes the reference signal. For the design of the DC-link voltage regulator and adaptive filter, the system parameters used are as follows:

(25) Vm=112 V–170 V,ωe=250 rad/s–533 rad/s,vdc=230 V–270 V,Lf=4 mH,rf=22 mΩ,Cdc=2200 μF.

The reference currents, control law, and PWM signals are calculated by the digital signal processor- (DSP-) based controller. The following section discusses the experimental evaluation of the proposed system.

## 4. Experimental Results

**Figure 6:**
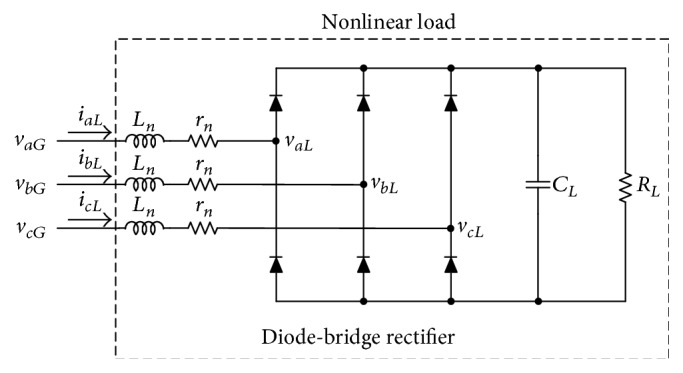
Power circuit of nonlinear load in the proposed system.

To verify the performance of the proposed system, a 2 kVA three-phase APF was developed, and the nonlinear load of Figure 6 was used with the following parameters:

(26) Ln=3 mH,rn=19 mΩ,CL=3300 μF,RL=9.6 Ω.

**Figure 7:**
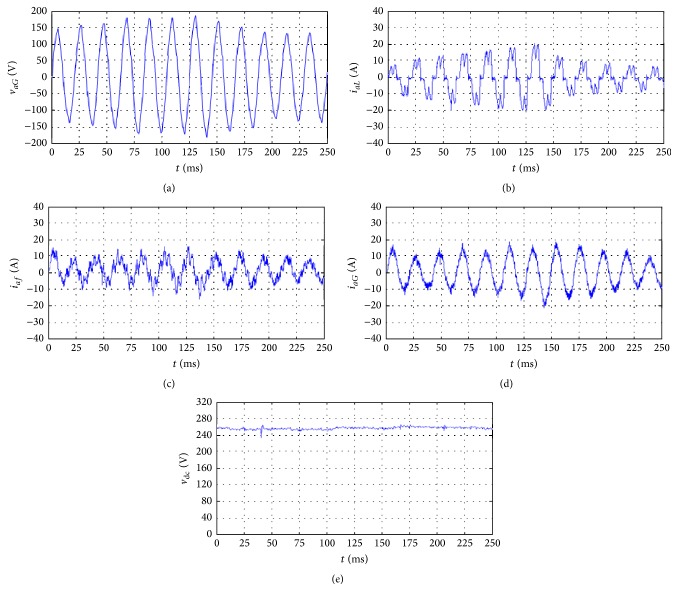
Experimental results of the proposed system with balanced nonlinear load: (a) phase voltage *v*_*aG*_ of the wind generator; (b) nonlinear load current *i*_*aL*_; (c) active power filter current *i*_*af*_; (d) line current *i*_*aG*_ of the wind generator; (e) DC-link voltage *v*_dc_.

The proposed system was experimentally evaluated using balanced and unbalanced nonlinear loads.  Figure 7 shows the phase voltage *v*_*aG*_ of the wind generator, nonlinear load current *i*_*aL*_, APF current *i*_*af*_, line current *i*_*aG*_ of the wind generator, and DC-link voltage *v*_dc_ for the balanced nonlinear load. As shown in the figure, when the wind speed varies between 30 and 40 m/s, the voltages and currents from the wind generator are in-phase and *i*_*aG*_ is almost sinusoidal, while the DC-link voltage *v*_dc_ remains constant. According to the experimental results, THD_*i*_ of *i*_*aG*_ is reduced from 18.63% (without an APF) to 3.68% when an APF is used. The power factor on the generator side increases from 0.84 (without an APF) to unity when an APF is used.

**Figure 8:**
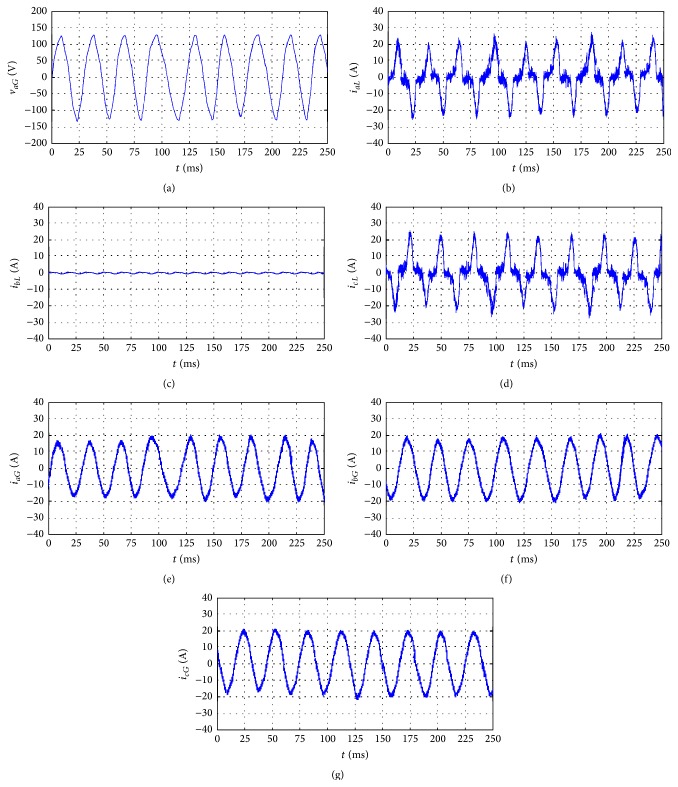
Experimental results of the proposed system with unbalanced nonlinear load: (a) phase voltage *v*_*aG*_ of the wind generator; (b) nonlinear load current *i*_*aL*_; (c) nonlinear load current *i*_*bL*_; (d) nonlinear load current *i*_*cL*_; (e) line current *i*_*aG*_ of the wind generator; (f) line current *i*_*bG*_ of the wind generator; (g) line current *i*_*cG*_ of the wind generator.

As indicated, the case of unbalanced load was examined by disconnecting the pair of diodes in phase *b* of Figure 6, which yielded *i*_*bL*_ = 0 and *i*_*cL*_ = −*i*_*aL*_. THD_*i*_ values calculated from Figure 8 for phases *a* and *c* were identical and equal to 31.3% with a power factor of 0.38 in the absence of an APF when the wind speed varied between 30 and 40 m/s. After using the APF, THD_*i*_ for phases* a*,* b*, and *c* became 7.01%, 3.11%, and 4.23%, respectively, with a unity power factor.

**Figure 9:**
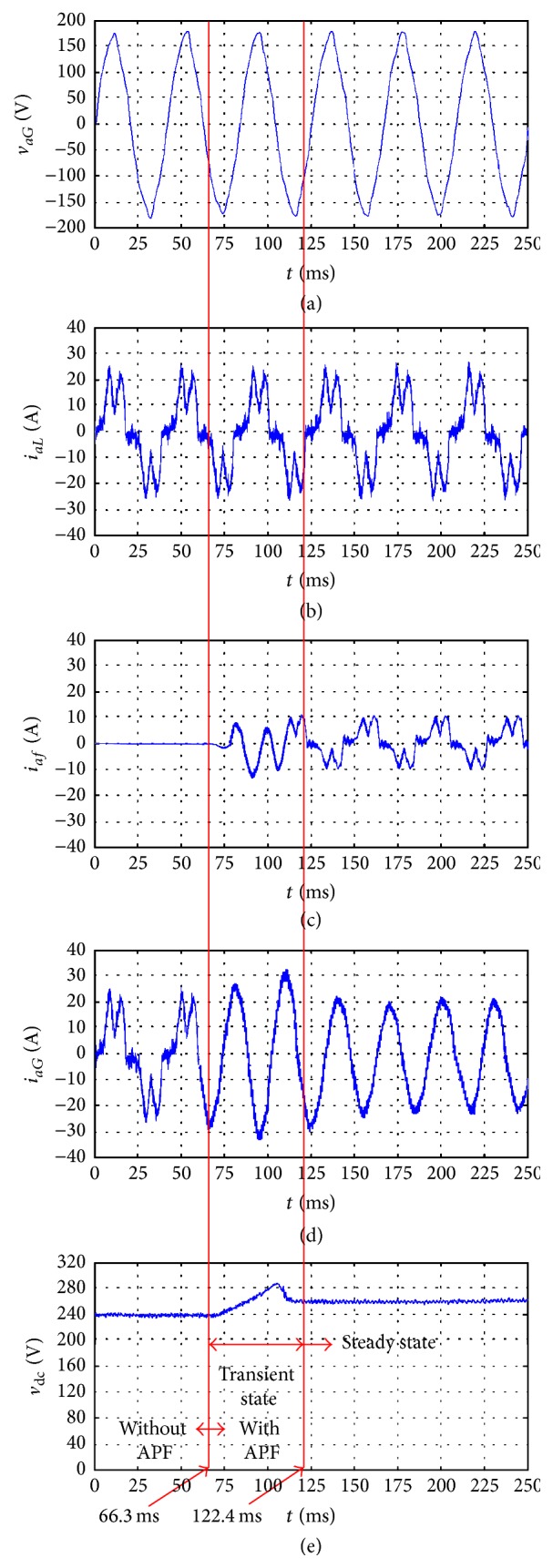
Experimental results of transient response of the proposed system with balanced nonlinear load: (a) phase voltage *v*_*aG*_ of the wind generator; (b) nonlinear load current *i*_*aL*_; (c) active power filter current *i*_*af*_; (d) line current *i*_*aG*_ of the wind generator; (e) DC-link voltage *v*_dc_.

Figure 9 shows the transient response in the experiment when the wind speed was fixed. As illustrated in Figure 9, the transient period for the DC-link voltage *v*_dc_ and line current of the wind generator is 56.1 ms. The proposed system demonstrates fast dynamic response for both compensating the current harmonics and establishing the DC-link voltage. The experimental results indicate that the proposed system can compensate current harmonics effectively and maintain excellent regulation of the DC-link voltage for balanced and unbalanced loads.

## 5. Conclusions

This paper presents the implementation and analysis of DSP-based digital control for a shunt-type three-phase APF with adaptive control for wind generators. With the proposed method, an APF can be used for suppressing current harmonics and compensating reactive power from loads. The novel adaptive low-pass filter is effective in reducing the phase delay for reference current calculations and DC-link voltage feedback. In addition, it can reduce the size of the DC-link capacitor and overall system cost to meet the requirements of industrial applications. Regardless of the wind speed variation, the design and analysis method is suitable for both balanced and unbalanced loads. The resulting THD_*i*_ of the line current from the wind generator is low, and a power factor of unity and effective regulation of the DC-link voltage can be achieved.
